# Molecular characterization of velogenic viscerotropic Ranikhet (Newcastle) disease virus from different outbreaks in desi chickens

**DOI:** 10.14202/vetworld.2017.319-323

**Published:** 2017-03-17

**Authors:** V. S. Dhaygude, G. K. Sawale, M. M. Chawak, N. R. Bulbule, S. D. Moregaonkar, D. S. Gavhane

**Affiliations:** 1 Department of Veterinary Pathology, Bombay Veterinary College, Parel, Mumbai, Maharashtra, India; 2 Department of Veterinary Pathology, Poultry Diagnostic and Research Centre, Lono Kalbhor, Pune, Maharashtra, India

**Keywords:** mean death time, Newcastle disease virus, reverse transcriptase polymerase chain reaction, specific pathogen free, velogenic

## Abstract

**Aim:**

Diagnosis of velogenic viscerotropic Ranikhet disease from six different flocks of desi chicken in and around Mumbai by gross and histopathological examination, isolation of virus and molecular methods.

**Materials and Methods:**

A total of 25 carcasses (varying between 2 and 6 carcasses from each flock) of six different flocks of adult desi chicken were subjected to necropsy examination for diagnosis of the disease during the span of a year (2014-2015). After thorough gross examination, the tissue samples were collected and processed for virus isolation and histopathological examination. The 20% tissue homogenate was inoculated into 9-day-old specific pathogen free (SPF) embryonated eggs. Mean death time (MDT) of embryos after inoculation and intracerebral pathogenicity index (ICPI) were used to judge velogenic nature of the virus. Newcastle disease virus (NDV) was isolated from six cases and confirmed by reverse transcriptase-polymerase chain reaction (RT-PCR) targeting the partial fusion protein gene of the viral genome.

**Results:**

A total of 25 carcasses (varying between 2 and 6 carcasses from each flock) of six different flocks of desi chicken were presented for postmortem examination to Department of Veterinary Pathology, Bombay Veterinary College, Parel, Mumbai during 2014-2015. The gross and histopathological examination revealed lesions suggestive of viscerotropic velogenic form of the Newcastle disease (ND). The 20% tissue homogenate was inoculated into 9-day-old embryonated eggs from SPF chicken. NDV was isolated from six cases and confirmed by RT-PCR targeting the partial fusion protein gene. MDT of all the isolates was <60 h which indicated velogenic nature of the virus. ICPI of the isolates ranged between the 1.63 and 1.78. In four out of six outbreaks concurrent moderate to heavy infection of *Ascardii galli* in one flock and *Railetina* spp. in three flocks was also noted. In this study, viscerotropic velogenic form of ND was confirmed in all six outbreaks by gross and microscopic examination, virus isolation and RT-PCR.

**Conclusions:**

In this study, viscerotropic velogenic form of ND was confirmed in all six outbreaks by gross and microscopic examination, virus isolation and RT-PCR. Nowadays, vaccine strains Lasota, B1 and F strains are used widely in India to control the infection of NDV. However, virulent NDV strains are still isolated frequently in the birds under backyard and also in commercial venture which demonstrates that NDV remains an on-going threat to commercial as well as backyard poultry flocks.

## Introduction

Newcastle disease (ND) is economically important and highly infectious viral disease of birds caused by virulent strains of avian paramyxovirus Type 1 of the genus Avulavirus belonging to the family *Paramyxoviridae*. The severity of disease produced varies with both host and strain of virus. The disease, on the basis of virulence and clinical signs, is characterized into different pathotypes, viz., viscerotropic velogenic (Doyle’s form), neurotropic velogenic (Beach’s form), mesogenic (Beudette’s form), lentogenic (Hitchner’s form), and asymptomatic enteric form [1]. The morbidity and mortality in a flock varies according to the strain involved, spanning from peracute disease with almost 100% mortality to subclinical disease with no lesions [2] and no mortality as well.

The genome of ND virus (NDV) has been predicted to have three genome lengths 15,186, 15,192, and 15,198 nucleotides [3] and encodes for six structural proteins, viz., fusion (F), nucleoprotein (NP), matrix (M), phosphoprotein (P), RNA polymerase (L), and hemagglutinin-neuraminidase [4]. The virulence of NDV is dependent on various factors, and the amino acid composition of the F protein cleavage site is the main determinant of NDV virulence and tissue tropism [5]. The pathogenicity of NDV can be determined on the basis of mean death time (MDT) in 9-10 days old embryonated chicken eggs (ECEs), intravenous pathogenicity index in 6-week-old chickens and intracerebral pathogenicity index (ICPI) in day-old chickens [6]. In the recent past, molecular techniques like polymerase chain reaction (PCR) have been frequently used worldwide to detect NDV in the field samples [7-9].

The disease is present worldwide and affects many species of birds causing severe losses to the poultry sector [4]. ND remains endemic in commercial poultry despite intensive vaccination programs that have been applied since the 1950s and major constraints to the development of both industrial and backyard poultry production in Asia.

In the present investigations, we have isolated and characterized viscerotropic velogenic NDV from outbreaks of six different locations in and around Mumbai during 2014-2015.

## Materials and Methods

### Ethical approval

The ethical approval is not necessary for such type of study. We have used the carcasses of chickens which were presented for postmortem examination.

### Farm history and sample collection

Carcasses from six flocks of desi chicken were presented for postmortem examination to the Department of Veterinary Pathology, Bombay Veterinary College, Parel, Mumbai, during the span of a year (2014-2015). The information on breed, flock size, age, clinical signs, morbidity and mortality was obtained from the owners for four flocks at the time of postmortem examination at the Department of Veterinary Pathology, Bombay Veterinary College and by personal visits for two flocks. The dead birds were subjected to detailed necropsy examination to note lesions.

### Histopathology

The tissues samples of proventriculus, portion of intestine, cecal tonsils, spleen, trachea, and lungs were collected in 10% neutral buffered formalin for histopathological examination. The tissues were processed by routine paraffin embedding technique and were stained by hematoxylin and eosin method of staining as per method suggested by Kiernan [10].

### Viral isolation

Tissue samples collected in 50% glycerine phosphate buffer saline (spleen, brain, trachea, and lung) from each flock were pooled and finely triturated, 20% suspension was prepared in phosphate buffered saline containing streptomycin (HiMedia make) at 2 mg/ml and penicillin (HiMedia make) at 2000 IU/ml. The suspension was cleared by centrifugation at 2000 rpm for 15 min. A volume of 0.2 ml of supernatant was injected into the allantoic cavity of 5, 9-day-old specific pathogen free (SPF) chicken eggs (Venkys India Ltd., Pune) and incubated at 37°C for 6 days. The eggs were subjected to candling twice daily. Eggs containing dead embryos were chilled to 4°C for overnight, and the allontoic fluid was harvested and tested for the presence of virus by hemagglutination assay using 1% chicken red blood cell and reverse transcriptase PCR (RT-PCR) assay.

### MDT

Serial 10-fold dilution of allantoic fluid (10-1 to 10-10) was prepared with sterile phosphate buffer saline and 0.1 ml of each dilution was inoculated into 5, 9-day-old SPF embryonated eggs (Venkys India Ltd., Pune) by allantoic route and then eggs were incubated at 37°C. The eggs were examined twice daily for maximum period of 6-day by candling the eggs and observations on death of embryos were recorded. The highest dilution at which all embryos died was taken as mean lethal dose (MLD), and the MDT was calculated as the average time at which the embryonated eggs inoculated with MLD were died [1].

### ICPI

The ICPI was determined by inoculating 0.05 ml of a 1:10 dilution of infective, bacteria free allantoic fluid in sterile isotonic saline into the brains of each of 10, 1-day-old SPF chicks. The birds were observed daily for 8 days, and the ICPI values were determined by using standard protocols [6,11].

### RNA isolation and RT-PCR amplification

Viral RNA was extracted from allantoic fluid using an RNA extraction kit (Ambion Life Technology) by Trizol Method and further RNA was used as a template for RT-PCR amplification. The forward primer 5’-CCTCATCCCAGACAGG 3 and reverse primer 5’-AAAGTTATCCCGTCTAAGGATAA 3 yielding 1148 bp product size were used for amplification of partial fusion protein gene of NDV. RT-PCR reaction was set up in a total volume of 50 µl per reaction. The reaction mixture was comprised of 25 µl one-step RT-PCR ×2 reaction mixture (buffer containing 0.4 mM of each deoxynucleotide triphosphates, 3.2 mM MgSO_4_, Invitrogen Superscript III one step RT-PCR system with platinum taq polymerase), 1 µl (10 pmol) each of the forward and reverse primers, 5 µl sample RNA, and 17 µl nuclease-free water. The steps in RT-PCR comprised synthesis of cDNA using a reverse transcription enzyme at 48°C for 30 min followed by initial denaturation (at 94°C for 2 min), 40 cycles of denaturation (94°C for 15 s) annealing (52°C for 30 s), extention (68°C for 1 min), and single cycle of final extension at 68°C for 5 min. For positive control, the viral RNA was extracted from live ND vaccine (Lasota) and for negative control nontemplate control were used. PCR products were visualized on a 1% agarose gel with ethidium bromide and detected under an ultraviolet transilluminator.

## Results and Discussion

### Gross and microscopic pathology

**Figure-1:**
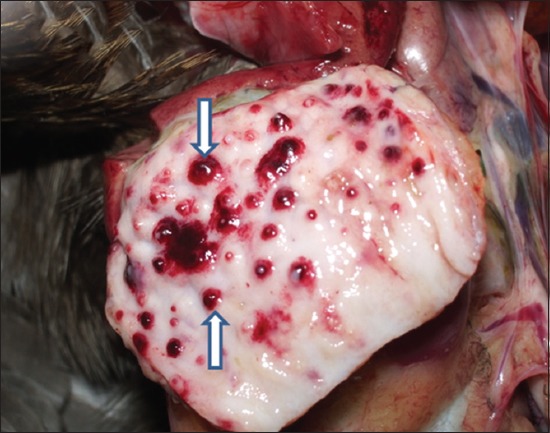
Gross picture showing hemorrhages at the tip of proventricular glands (arrows with up and down heads).

**Figure-2:**
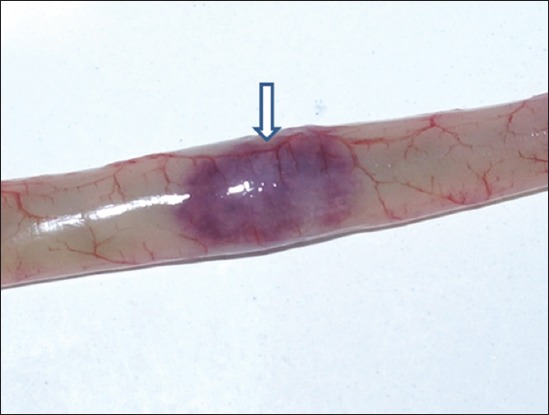
Hemorrhagic ulcers in intestine seen through serosa (arrow with down head).

**Figure-3:**
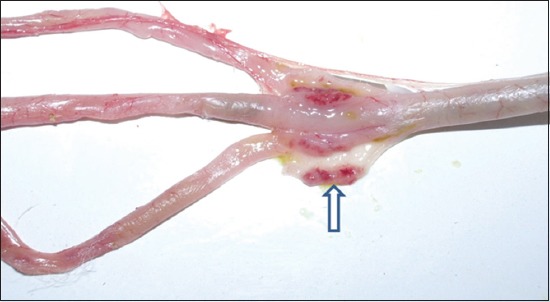
Hemorrhages in cecal tonsils (arrow with up head).

History revealed sudden onset of disease in the flocks of adult desi chicken (Aseel breed) with mortality ranging between 70% and 90%. Most of the birds died suddenly without preceding signs. The necropsy examination of the majority of dead birds revealed hemorrhages at the tips of proventricular glands (Figure-1), multifocal hemorrhages evident through serosal surface of the intestines with typical button ulcers filled in with debris/blood and exudate in intestinal mucosa (Figure-2) and cecal tonsils hemorrhages (Figure-3) as a common lesions Spleen was enlarged and showed hemorrhages with focal areas of necrosis in most of the cases. Trachea revealed congestion and petechial hemorrhages. The lung lesions were noted in few birds and consisted of hemorrhages, congestion, emphysema, and edema. All above lesions were noted in most of the necropsied birds with little variation in the frequency of occurrence of lesions. Similar gross pathological changes were observed by Hamid *et al*. [5] and Wakamatsu *et al*. [12] in velogenic viscerotropic ND (VVND). Concurrent moderate to heavy infection of *Ascardii galli* in one flock and *Railetina* spp. in three flocks was noted.

**Figure-4:**
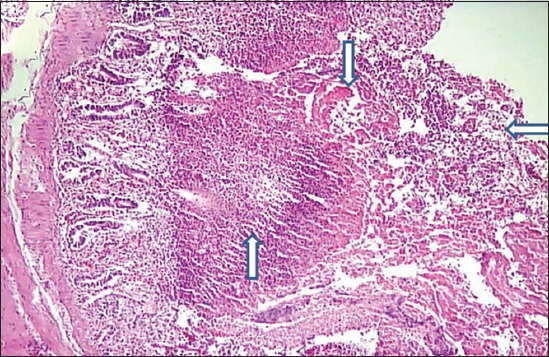
Section of intestine showing extensive hemorrhages (arrow with down head), multifocal necrosis (arrow with up head), loss of epithelial cells lining villi and infiltration of inflammatory cells (arrow with horizontal head). (H and E, 100×).

**Figure-5:**
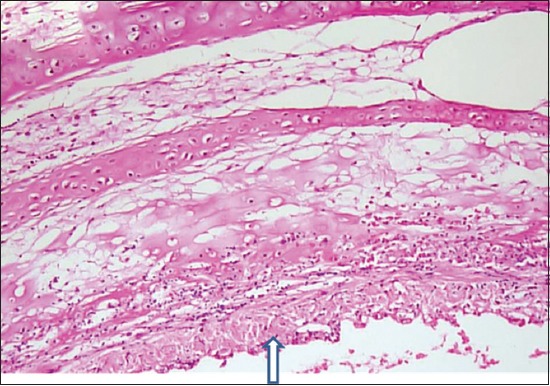
Section of trachea with necrosis of tracheal epithelial cell along with mild infiltration of inflammatory cells (arrow with up head), congestion of vessels and edema in submucosa. (H and E, 100×).

Histopathologically, hemorrhages of varying severity involving tips of proventricular glands were noted in the majority of birds. There were hemorrhages, multifocal necrosis, loss of epithelial cells lining villi (Figure-4), and ulceration of gut-associated lymphoid tissue where the lymphoid aggregates were replaced by necrotic debris and fibrin in the intestine as common finding. The area underlying ulcer showed infiltration of inflammatory cells predominantly polymorphonuclear cells. Hemorrhagic and necrotic lesions were seen in the cecal tonsil. The spleen showed severe depletion of lymphocytes in splenic follicles with multifocal areas of necrosis and apoptosis and hemorrhages along with infiltration of inflammatory cells in the parenchyma (data not shown as a figure). Trachea from many birds showed multifocal necrosis of tracheal epithelial cell along with mild infiltration of inflammatory cells, congestion of vessels and edema in submucosa (Figure-5). The lesions suggested VVND as similar lesions were described by Hamid *et al*. [5] and Wakamatsu *et al*. [12] in VVND affected chicken.

### Virus isolation, RT-PCR, and pathogenicity of NDV

**Figure-6:**
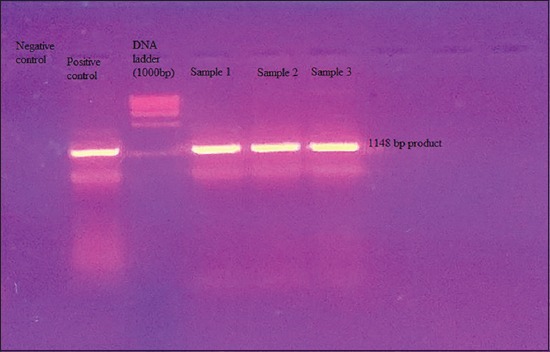
Reverse transcriptase polymerase chain reaction amplification of the fusion protein gene of RD virus (1148 bp product) from three cases.

The study is focused on isolating the virus from field condition and characterizes them at biological level. The suspension from feces and pooled organ samples (trachea, spleen, lung, and brain) was inoculated into the 9-day-old embryonated SPF chicken eggs by allantoic route. All the six samples were confirmed as ND based on virus isolation and RT-PCR. The RT-PCR amplification of the partial fusion protein gene of the NDV revealed 1148 bp product for all the samples (Figure-6). RT-PCR technique has been routinely used for diagnosis of NDV and detecting NDV in clinical samples without necessitating virus isolation in ECE [13,14]. Singh *et al*. [9] also used RT-PCR for detection of ND viral genome directly from the clinical samples from the field outbreaks in chicken in India. Pathogenicity testing by the MDT and ICPI tests showed that all the isolates were of velogenic pathotype. Isolates from all the six outbreaks caused death of embryos within 60 h of inoculation indicating velogenic nature of the virus. The ICPI values were 1.67, 1.71, 1.64, 1.78, 1.8, and 1.63 for six different isolates. ICPI is the most reliable and reproducible test providing a good indication of relative virulence of the viruses [4]. The ICPI of all the isolates ranged from 1.63 to 1.78. These values are in accordance with previous reports and OIE guidelines.

Nowadays, vaccine strains Lasota, B1 and F strains are used widely in India to control the infection of NDV. However, virulent NDV strains are still isolated frequently in the vaccinated and unvaccinated birds, which demonstrate that NDV remains an on-going threat to backyard as well as commercial poultry flocks. The outbreaks in backyard venture are potential source of infection to commercial chickens. The vaccination and other biosecurity measures are still neglected by communities rearing chicken under backyard venture in India; hence, there is need to educate people on these issues.

Furthermore, gastrointestinal infection of nematodes and cestodes suggests negligence of deworming in desi chicken in backyard venture. Gastrointestinal parasitism adversely affects health and also reduces efficacy of vaccines. This indicates need of extensive efforts to create awareness among poultry owners about importance of deworming in maintaining health of the birds.

## Conclusions

In this study, VVND was confirmed in all six outbreaks by gross and microscopic examination, virus isolation and RT-PCR. Nowadays, vaccine strains Lasota, B1 and F are used widely in India to control the infection of NDV. However, virulent NDV strains are still isolated frequently in the birds under backyard and also in commercial venture which demonstrates that NDV remains an on-going threat to commercial as well as backyard poultry flocks. This also presses on need to create awareness regarding the importance of vaccination and other biosecurity measures for control of ND. Furthermore, these outbreaks can be a potential source of infection to chickens under commercial set up.

## Authors’ Contributions

VSD, GKS, DSG: Research was designed and executed by these authors as a part of diagnostic services to the poultry farmers. Postmortem, gross, histopathological examination, sample collection, photography, and interpretation of related data were done by these authors. SDM: Supervised the work and helped in manuscript preparation. MMC and NRB contributed in virus isolation, RT-PCR and determination of MDT and ICPI and also done interpretation of the related data. All authors read and approved the final manuscript.
